# 

**Published:** 1875-05

**Authors:** 


### CONTENTS:

ORIGINAL COMMUNICATIONS:

ART. I. Various Uses of Chloride of Zinc. By J. E. Nichols, M.D. ....321

ART. II. Koumiss as a Remedial Agent—Report of Cases. By R. L Leonard, M.D. ....326

PROGRESS IN MEDICAL SCIENCES:

ART. I. Progress of Obstetrics. By E. Warren Sawyer, M.D. ....330

Art. II. Progress of Gynæcology. By A. Reeves Jackson, M.D. ....334

SELECTIONS.

Impressions of American Surgery ....340

Case of Aneurism of the Abdominal Aorta ....353

A Strychnia Eater, etc ....354

Spinal Arthropathies ....355

Similarity between Red Blood-Corpuscles of Man and those of certain other Mammals, etc ....376

Explanatory Note in Regard to Diagnosis of Blood Stains ....390

REPORTS OF SOCIETIES ....368

EDITORS' BOOK TABLE ....393

MORTALITY STATISTICS, Chicago ....398

### *Illinois State Medical Society.*

The Twenty-fifth Annual Session will be held in the city of Jacksonville, on the third Tuesday in May (18th), 1875, at 9 o'clock, A. M. The following Committees are expected to report :

Committee on Practical Medicine — E. P. Cook, M.D., Mendota, *Chairman.*
Committee on Surgery — Edwin Powell, M.D., Chicago, *Chairman.*
Committee on Obstetrics — Hiram Nance, M.D., Kewanee, *Chairman.*
Committee on Drugs and Medicines — Wm. E. Quine, M.D., Chicago, *Chairman.*
Committee on Necrology — E. Ingals, M.D., Chicago, *Chairman.*
Committee on Opthalmology — E. L. Holmes, M.D., Chicago, *Chairman.*
Committee on Otology — S. J. Jones, M.D., Chicago, *Chairman.*

SPECIAL COMMITTEES.

Committee on Diseases of Children — J. O. Hamilton, M.D., Jerseyville, *Chairman.*
Committee on Diseases of Respiratory Organs — H. A. Johnson, M.D., Chicago, *Chairman.*
Committee on Galvano-Therapeutics — David Prince, M.D., Jacksonville, *Chairman.*
Committee on Diseases of Women — J. T. Pearman, M.D., Champaign, *Chairman.*
Committee on Diseases of the Nervous System — J. S. Whitmire, M.D., Metamora, *Chairman.*
Committee on Medical Jurisprudence — R. J. Patterson, M.D., Batavia, *Chairman.*
Committee on Dermatology — Chas. G. Smith, M.D., Chicago, *Chairman.*
Committee on Public Address — J. H. Hollister, M.D., Chicago, *Ch'n.*

Each local Society shall have the privilege of sending to the Society one delegate for every five of its regular resident members, and one for every additional fraction of more than half of this number. The faculty of every regularly constituted medical college or chartered school of medicine, shall have the privilege of sending two delegates. The professional staff of every chartered or municipal hospital, and every other permanently organized medical institution of good standing, shall have the privilege of sending one delegate.

*The Members by Invitation* shall consist of practitioners of reputable standing from any part of the United States. They shall receive their appointment by invitation of the meeting, after an introduction from any of the members present, or from any of the absent permanent members. They shall hold their connection with the Society until the close of the session at which they were received, and may participate in the discussions without the right of voting.

*The Permanent Members* shall consist of regular graduates from reputable schools of medicine, who have served in the capacity of delegates, or such other physicians as may be proposed by two members of this Society, reported on favorably by the Committee of Investigation, and receiving a two-thirds vote of all the members present.

A circular will be sent to each member, from the Committee of Arrangements, giving all necessary information relative to the programme and the arrangements made with the different railroads in regard to reduction of fare, etc.

T. DAVIS FITCH, M.D., *Permanent Secretary,*
296 W. Monroe St., Chicago.

